# Predicting Consensus Structures for RNA Alignments Via Pseudo-Energy Minimization

**DOI:** 10.4137/bbi.s2578

**Published:** 2009-06-03

**Authors:** Junilda Spirollari, Jason T.L. Wang, Kaizhong Zhang, Vivian Bellofatto, Yongkyu Park, Bruce A. Shapiro

**Affiliations:** 1 Bioinformatics Program, Department of Computer Science, New Jersey Institute of Technology, University Heights, Newark, NJ 07102, U.S.A; 2 Department of Microbiology and Molecular Genetics, University of Medicine and Dentistry of New Jersey-New Jersey Medical School, International Center for Public Health, 225 Warren Street, Newark, NJ 07103, U.S.A; 3 Department of Computer Science, University of Western Ontario, London, Ontario, N6A 5B7, Canada; 4 Department of Cell Biology and Molecular Medicine, University of Medicine and Dentistry of New Jersey-New Jersey Medical School, Newark, NJ 07103, U.S.A; 5 Center for Cancer Research Nanobiology Program, National Cancer Institute, Frederick, MD 21702, U.S.A

**Keywords:** RNA secondary structure prediction, *Drosophila* secondary structure, Rfam sequence alignments, normalized energy

## Abstract

Thermodynamic processes with free energy parameters are often used in algorithms that solve the free energy minimization problem to predict secondary structures of single RNA sequences. While results from these algorithms are promising, an observation is that single sequence-based methods have moderate accuracy and more information is needed to improve on RNA secondary structure prediction, such as covariance scores obtained from multiple sequence alignments. We present in this paper a new approach to predicting the consensus secondary structure of a set of aligned RNA sequences via pseudo-energy minimization. Our tool, called RSpredict, takes into account sequence covariation and employs effective heuristics for accuracy improvement. RSpredict accepts, as input data, a multiple sequence alignment in FASTA or ClustalW format and outputs the consensus secondary structure of the input sequences in both the Vienna style Dot Bracket format and the Connectivity Table format. Our method was compared with some widely used tools including KNetFold, Pfold and RNAalifold. A comprehensive test on different datasets including Rfam sequence alignments and a multiple sequence alignment obtained from our study on the *Drosophila* X chromosome reveals that RSpredict is competitive with the existing tools on the tested datasets. RSpredict is freely available online as a web server and also as a jar file for download at http://datalab.njit.edu/biology/RSpredict.

## Introduction

RNA secondary structure prediction has been studied for quite awhile. Many minimum free energy (MFE) methods have been developed for predicting the secondary structures of single RNA sequences, such as mfold,^1^ RNAfold,^2^ MPGAfold,^3^ as well as recent tools presented in the literature.^4^ However, the accuracy of predicted structures is far from perfect. Recently, a new concept of normalized free energy for predicting the secondary structures of single RNA sequences was introduced.^5^ The normalized free energy of an RNA substructure is the free energy of that substructure divided by the length of its underlying sequence. A dynamic programming algorithm, called Densityfold, was developed, which delocalizes the thermodynamic cost of computing RNA substructures and improves on secondary structure prediction via normalized energy minimization.^5^ Here, we extend the concept used in Densityfold and present a new tool, called RSpredict, for RNA secondary structure prediction. RSpredict computes the RNA structure with minimum normalized energy based on the loop decomposition scheme used in the nearest neighbor energy model.^6^–^8^ The new tool focuses on the loops in an RNA secondary structure, whereas Densityfold considers RNA substructures where a substructure may contain several loops.

**Figure 1.**
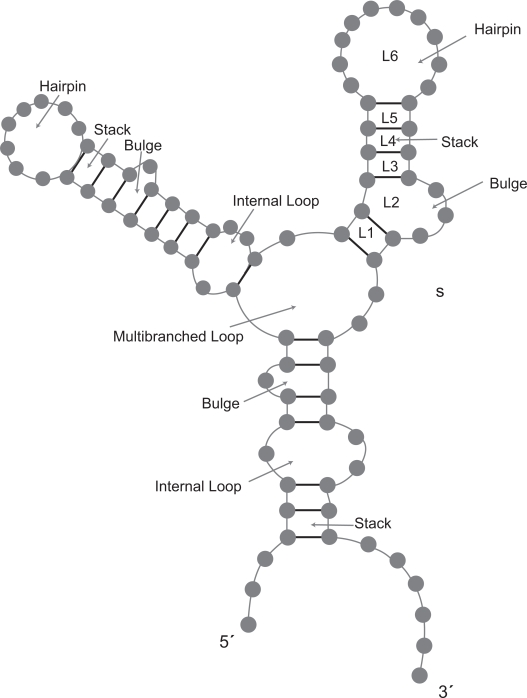
Illustration of the loops and substructures in an RNA secondary structure. Each loop has at least one base pair. A stem consists of two or more consecutive stacks shown in the figure.

To understand the difference between the two tools, see Figure 1. In the figure, the substructure *S* contains six loops, denoted as *L*_1_, *L*_2_, *L*_3_, *L*_4_, *L*_5_, *L*_6_. RSpredict calculates the normalized free energy of the substructure *S*, denoted *NE*(*S*), by taking the sum of the normalized energies of the six loops. That is,

$$NE(S)=\sum_{i=1}^{6}NE(Li)$$

where

$$NE(L_{i})=\frac{E(L_{i})}{\left|L_{i}\right|}$$

*E*(*L_i_*) is the free energy of loop *L_i_* and |*L_i_*| is the length of *L_i_*, i.e. the number of nucleotides in *L_i_*. On the other hand, Densityfold computes the normalized free energy of the substructure *S* as the sum of normalized energies of all its substructures including S. Referring to Figure 1, we see that *S* has six substructures, denoted as *S*_1_ = *S*, *S*_2_, *S*_3_, *S*_4_, *S*_5_, *S*_6_, where *S*_1_ = {*L*_1_, *L*_2_, *L*_3_, *L*_4_, *L*_5_, *L*_6_}, *S*_2_ = {*L*_2_, *L*_3_, *L*_4_, *L*_5_, *L*_6_}, *S*_3_ = {*L*_3_, *L*_4_, *L*_5_, *L*_6_}, *S*_4_ = {*L*_4_, *L*_5_, *L*_6_}, *S*_5_ = {*L*_5_, *L*_6_}, *S*_6_ = {*L*_6_}. Therefore the normalized energy of the substructure *S* is

$$NE(S)=\sum_{i=1}^{6}NE(S_{i})$$

where

$$NE(S_{i})=\frac{E(S_{i})}{\left|S_{i}\right|}$$

*E*(*S_i_*) is the free energy of substructure *S_i_* and |*S_i_*| is the number of nucleotides in *S_i_*. The difference in the normalized energy computation leads to a new algorithm for RSpredict, which is different from the algorithm used by Densityfold.

While the normalized energy model creates a foundation for RNA secondary structure prediction, there are many limitations in Densityfold, just like in all other single sequence-based MFE methods. Optimal structures predicted by these methods do not necessarily represent real structures. This happens due to several reasons. The thermodynamic model may not be accurate. The bases of structural RNAs may be chemically modified and these processes are not included in the prediction model. Finally, some functional RNAs may not have stable secondary structures. Thus, a more reliable approach is to use comparative analysis to compute consensus secondary structures from multiple related RNA sequences.

In general, there are three strategies with the comparative approach. The first strategy is to predict the secondary structures of individual RNA sequences separately and then align the structures. Tools such as STRUCTURELAB^9^ and RADAR^6^ are based on this strategy. The second strategy predicts common secondary structures of two or more RNA sequences through simultaneous alignment and consensus structure inference. Tools based on this strategy include RNAscf,^10^ Dynalign,^11^ stemloc,^12^ and CARNAC.^13^ These tools utilize either folding free energy change parameters or stochastic context-free grammars (SCFGs) and are considered derivations of Sankoff’s method.^14^

The third strategy is to fold multiple sequence alignments. RNAalifold^15^,^16^ uses a dynamic programming algorithm to compute the consensus secondary structure with minimum free energy by taking into account thermodynamic stability, sequence covariation together with RIBOSUM-like scoring matrices. Pfold^17^ is a SCFG algorithm that produces a prior probability distribution of RNA structures. A maximum likelihood approach is used to estimate a phylogenetic tree for predicting the most likely structure for input sequences. A limitation of Pfold is that it does not run on alignments of more than 40 sequences and in some cases produces no structures due to under-flow errors. Maximum weighted matching (MWM), based on a graph-theoretical approach and developed by Cary and Stormo^18^ and Tabaska et al,^19^ is able to predict common secondary structures allowing pseudoknots. KNetFold^20^ is a recently published machine learning method, implemented using a hierarchical network of k-nearest neighbor classifiers that analyzes the base pairings of alignment columns in the input sequences through their mutual information, Watson-Crick base pairing rules and thermodynamic base pair propensity derived from RNAfold.^2^ The method proposed in this paper, RSpredict, joins the many tools using the third strategy; it accepts a multiple alignment of RNA sequences as input data and predicts the consensus secondary structure for the input sequences by minimizing their pseudo-energy, which takes into account both the normalized free energy and covariance scores of the input sequences.

The rest of the paper is organized as follows. We first describe the implementation and algorithms used by RSpredict, and analyze the time complexity of the algorithms. We then present experimental results of running the RSpredict tool as well as comparison with the existing tools. The experiments were performed on a variety of datasets. Finally we discuss some properties of RSpredict, possible ways to improve the tool and point out some directions for future research.

## Methods

RSpredict, which can be freely downloaded from http://datalab.njit.edu/biology/RSpredict, was implemented in the Java programming language. The program accepts, as input data, a multiple sequence alignment in FASTA or ClustalW format and outputs the consensus secondary structure of the input sequences in both the Vienna style Dot Bracket format^16^ and the Connectivity Table format.^21^ Below, we describe the normalized energy model adopted by RSpredict. We then present a dynamic programming algorithm for folding a single RNA sequence via normalized energy minimization. Next, we describe techniques for calculating covariance scores based on the input alignment. Finally we summarize the algorithms used by RSpredict, combining both the folding technique and the covariance scores obtained from the input alignment, and show its time complexity.

### Folding of a single RNA sequence

We represent an RNA secondary structure as a fully decomposed set of loops.^6^–^8^ In general, a loop *L* can be one of the following (Fig. 1):

- a hairpin loop (which is a loop enclosed by only one base pair; the smallest possible hairpin loop consists of 3 nucleotides enclosed by a base pair);
- a stack, composed of two consecutive base pairs;
- a bulge loop, if two base pairs are separated only on one side by one or more unpaired bases;
- an internal loop, if two base pairs are separated by one or more unpaired bases on both sides;
- a multibranched loop, if more than two base pairs are separated by zero or more unpaired bases in the loop.

We now introduce some terms and definitions. Let *S* be an RNA sequence consisting of nucleotides or bases A, U, C, G. *S* [*i*] denotes the base at position *i* of the sequence *S* and *S* [*i*, *j*] is the subsequence starting at position *i* and ending at position *j* in *S*. A base pair between nucleotides at positions *i* and *j* is denoted as (*i*, *j*) or (*S* [*i*], *S* [*j*]), and its enclosed sequence is *S* [*i*, *j*]. Given a loop *L* in the secondary structure *R* of sequence *S*, the base pair (i*, j*) in *L* is called the *exterior pair* of *L* if *S* [i*] (*S* [j*], respectively) is closest to the 5′ (3′, respectively) end of *R* among all nucleotides in *L.* All other non-exterior base pairs in *L* are called *interior pairs* of *L.* The *length* of *L* is the number of nucleotides in *L.* Note that two loops may overlap on a base pair. For example, the interior pair of a stack may be the exterior pair of another stack, or the exterior pair of a hairpin loop. Also note that a bulge or an internal loop has exactly one exterior pair and one interior pair.

We use the normalized energy concept as follows. Given a secondary structure *R*, every base pair (*i*, *j*) in *R* is the exterior pair of some loop *L*. We assign (*i*, *j*) and *L* a normalized energy, which is the free energy of loop *L* divided by the length of *L*. The set of free energy parameters for non-multibranched loops used in our algorithm is acquired from Mathews et al.^8^ The free energy of a multibranched loop is computed based on the approach adopted by mfold,^1^ which is a linear function of the number of unpaired bases and the number of base pairs inside the loop, namely *a+b × n*_1_+*c ×n*_2_, where *a*, *b*, *c* are constants, *n*_1_ is the number of unpaired bases and *n*_2_ is the number of base pairs inside the multibranched loop. We adopt the loop decomposition scheme used in the nearest neighbor energy model developed by Mathews et al. 8 The secondary structure *R* contains multiple loop components and the normalized energies of the loop components are additive based on our definition of the normalized energy of a structure, as explained in the beginning of the Introduction section and illustrated in Figure 1. Our folding algorithm computes the total normalized energy of *R* by summing up all normalized energies of the loops in *R*. Thus, the RNA folding problem can be formalized as follows. Given an RNA sequence *S*, find the set of base pairs (*i*, *j*) and loops with (*i*, *j*) as exterior pairs, such that the total normalized energy of the loops (or equivalently, the exterior pairs) is minimized. The set of base pairs constitutes the optimal secondary structure of *S*.

**Figure 2.**
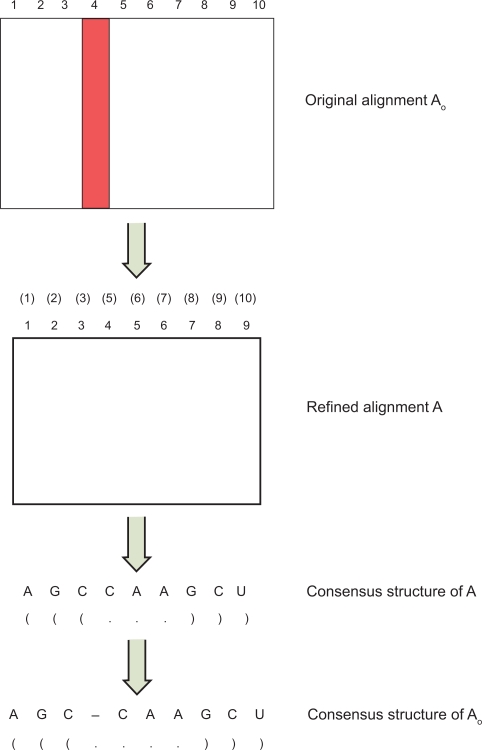
Illustration of the consensus structure definition used by RSpredict. Here, the RSpredict algorithm transforms the original input alignment *A*_o_ to a refined alignment *A* by deleting the fourth column (the column in red) of *A*_o_. The algorithm predicts the consensus structure of the refined alignment *A*. Then the algorithm generates the consensus structure of *A*_o_ by inserting an unpaired gap to the fourth position of the consensus structure of *A*. The numbers inside parentheses in the refined alignment *A* represent the original column numbers in *A*_o_.

When generalizing the folding of a single sequence to the prediction of the consensus structure of a multiple sequence alignment, we introduce the notion of refined alignments. At times, an input alignment may have some columns each of which contains more than 75% gaps. Some tools including RSpredict delete these columns to get a refined alignment;^17^ some tools simply use the original input alignment as the refined alignment. Suppose the original input alignment *A*_o_ has *N* sequences and *n*_o_ columns, and the refined alignment *A* has *N* sequences and *n* columns, *n* ≤ *n*_o_. Formally, the consensus structure of the refined alignment *A* is a secondary structure *R* together with its sequence *S* such that each base pair (*S* [*i*], *S* [*j*]), 1 ≤ *i* < *j* ≤ *n*, in *R* corresponds to the pair of columns *i*, *j* in the alignment *A*, and each base *S* [*i*], 1 ≤ *i* ≤ *n*, is the representative base of the *i*th column in the alignment *A*. There are several ways to choose the representative base. For example, *S* [*i*] could be the most frequently occurring nucleotide, excluding gaps, in the *i*th column of the alignment *A*. Furthermore, there is an energy measure value associated with each base pair (*S* [*i*], *S* [*j*]) or more precisely its corresponding column pair (*i*, *j*), such that the total energy measure value of all the base pairs in *R* is minimized. The consensus secondary structure of the original input alignment *A*_o_ is defined as the structure *R*_o_, obtained from *R*, as follows: (i) the base (base pair, respectively) for column *C*_o_ (column pair (*C*_o1_, *C*_o2_), respectively) in *A*_o_ is identical to the base (base pair, respectively) for the corresponding column *C* (column pair (*C*_1_, *C*_2_), respectively) in *A* if *C*_o_ ((*C*_o1_, *C*_o2_), respectively) is not deleted when getting *A* from *A_o_*; (ii) unpaired gaps are inserted into *R*, such that each gap corresponds to a column that is deleted when getting *A* from *A*_o_ (see Fig. 2).

In what follows, we first present an algorithm for folding a single RNA sequence based on the normalized energy concept described here. We then generalize the algorithm to predict the consensus secondary structure for a set of aligned RNA sequences.

#### Algorithm

The functions and parameters used in our algorithm are defined below where *S*[*i*, *j*] is a subsequence of *S* and *R* [*i*, *j*] is the optimal secondary structure of *S* [*i*, *j*]:

- *NE* (*i*, *j*) is the total normalized energy of all loops in *R*[*i*, *j*], where nucleotides at positions *i*, *j* may or may not form a base pair.
- *NE_P_* (*i*, *j*) is the total normalized energy of all loops in *R* [*i*, *j*] if nucleotides at positions *i*, *j* form a base pair.
- *e_H_* (*i*, *j*) (*E_H_* (*i*, *j*), respectively) is the free energy (normalized energy, respectively) of the hairpin with exterior pair (*i*, *j*).
- *e_s_* (*i*, *j*) (*E_S_* (*i*, *j*), respectively) is the free energy (normalized energy, respectively) of the stack with exterior pair (*i*, *j*) and interior pair (*i* + 1, *j* − 1).
- *e_B_* (*i*, *j*, *i*′, *j*′) (*E_B_* (*i*, *j*, *i*′, *j*′), respectively) is the free energy (normalized energy, respectively) of the bulge or internal loop with exterior pair (*i*, *j*) and interior pair (*i'*, *j'*).
- *e_J_* ( $i,j,i_{1}^{\prime },j_{1}^{\prime },i_{2}^{\prime },j_{2}^{\prime },\ldots ,i_{k}^{\prime },j_{k}^{\prime }$) *E_J_* ( $i,j,i_{1}^{\prime },j_{1}^{\prime },i_{2}^{\prime },j_{2}^{\prime },\ldots ,i_{k}^{\prime },j_{k}^{\prime }$), respectively) is the free energy (normalized energy, respectively) of the multi-branched loop with exterior pair (*i*, *j*) and interior pairs ( $i_{1}^{\prime },j_{1}^{\prime }$), ( $i_{2}^{\prime },j_{2}^{\prime }$), …,( $i_{k}^{\prime },j_{k}^{\prime }$).

It is clear that:

(1) $$E_{H}(i,j)=\frac{e_{H}(i,j)}{j-i+1}$$

(2) $$E_{s}(i,j)=\frac{e_{s}(i,j)}{4}$$

(3) $$E_{B}(i,j,i^{\prime },j^{\prime })=\frac{e_{B}(i,j,i^{\prime },j^{\prime })}{\left(i^{\prime }-i+j-j^{\prime }+2\right)}$$

(4) $$\begin{array}{l} E_{J}(i,j,{i^{\prime }}_{1},{j^{\prime }}_{1},{i^{\prime }}_{2},{j^{\prime }}_{2}\ldots ,{i^{\prime }}_{k},{j^{\prime }}_{k})\\ \;\;\;\;\;\;=\frac{e_{J}(i,j,{i^{\prime }}_{1},{j^{\prime }}_{1},{i^{\prime }}_{2},{j^{\prime }}_{2},\ldots ,{i^{\prime }}_{k},{j^{\prime }}_{k})}{n_{1}+2\times n_{2}} \end{array}$$

The denominator in Equation (2) is 4 because there are four nucleotides in the stack. In (4), *n*_1_ is the number of unpaired bases and *n*_2_ is the number of base pairs in the multibranched loop. Using Equations (1), (2), (3) and (4), the total normalized energy of all loops in *R*[*i*, *j*] where (*i*, *j*) is a base pair is computed by Equation (5):

(5) $$NE_{P}(i,j)=\text{min}\left\{\begin{array}{l} E_{H}(i,j)\\ E_{S}(i,j)+NE_{P}(i+1,j-1)\\ \underset{i<i^{\prime }<j^{\prime }<j}{\text{min}}\{E_{B}(i,j,i^{\prime },j^{\prime })+NE_{P}(i^{\prime },j^{\prime })\}\\ \underset{i<{i^{\prime }}_{1}<{j^{\prime }}_{1}<{i^{\prime }}_{2}<{j^{\prime }}_{2}<\ldots <{i^{\prime }}_{k}<{j^{\prime }}_{k}<j}{\text{min}}\left\{\begin{array}{l} E_{J}(i,j,{i^{\prime }}_{1},{j^{\prime }}_{1},{i^{\prime }}_{2},{j^{\prime }}_{2},\ldots {i^{\prime }}_{k},{j^{\prime }}_{k})\\ +\sum_{r=1}^{k}NE_{P}({i^{\prime }}_{r},{j^{\prime }}_{r}) \end{array}\right\} \end{array}\right\}$$

That is, the normalized energy *NE_p_* (*i*, *j*) is calculated by taking the minimum of the following four cases:

- (*i*, *j*) is the exterior pair of a hairpin, in which case the normalized energy *NE_P_* (*i*, *j*) equals *E_H_* (*i*, *j*), which is the normalized energy of the hairpin;
- (*i*, *j*) is the exterior pair of a stack, in which case *NE_P_* (*i*, *j*) equals the normalized energy of the stack, i.e. *E_S_* (*i*, *j*), plus *NE_P_* (*i* + 1, *j* − 1);
- (*i*, *j*) is the exterior pair of a bulge or an internal loop, in which case *NE_P_* (*i*, *j*) equals the minimum of the normalized energy of the bulge or internal loop *E_B_* (*i*, *j*, *i*′, *j*′) plus *NE_P_* (*i′, j′*) for all *i* < *i*′ < *j*′ < *j*;
- (*i*, *j*) is the exterior pair of a multibranched loop, in which case *NE_P_* (*i*, *j*) equals the minimum of the normalized energy of the multibranched loop *E* ( $i,j,i_{1}^{\prime },j_{1}^{\prime },i_{2}^{\prime },j_{2}^{\prime },\ldots ,i_{k}^{\prime },j_{k}^{\prime }$) plus $\sum_{r=1}^{k}NE_{P}(i_{r}^{\prime },j_{r}^{\prime })$, for all $i<i_{1}^{\prime }<j_{1}^{\prime }<i_{2}^{\prime }<j_{2}^{\prime }<\ldots <i_{k}^{\prime }<j_{k}^{\prime }<j$.

Equation (6) below shows the recurrence formula for calculating *NE*(*i*, *j*):

(6) $$NE(i,j)=\text{min}\left\{\begin{array}{l} NE(i,j-1)\\ NE(i+1,j)\\ NE_{P}(i,j)\\ \underset{i<k<j}{\text{min}}\{NE(i,k-1)+NE(k,j)\} \end{array}\right\}$$

**Figure 3.**
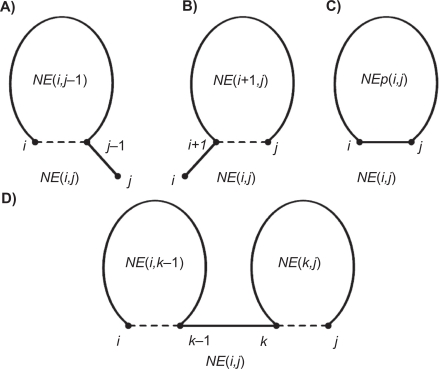
Illustration of the four cases in Equation (6). **A**) *NE*(*i*, *j*) equals *NE*(*i*, *j*−1) which is the total normalized energy of all loops in the optimal secondary structure *R*[*i*, *j* − 1] of subsequence *S*[*i*, *j* − 1]. **B**) *NE*(*i*, *j*) equals *NE*(*i* + 1, *j*) which is the total normalized energy of all loops in the optimal secondary structure *R*[*i* + 1, *j*] of subsequence *S*[*i* + 1, *j*]. **C**) *NE*(*i*, *j*) equals *NE_P_*(*i*, *j*) which is the total normalized energy of all loops in the optimal secondary structure *R*[*i*, *j*] of subsequence *S*[*i*, *j*], where *S*[*i*] and *S*[*j*] form a base pair. **D**) *NE*(*i*, *j*) equals the minimum of *NE*(*i*, *k* − *1*) plus *NE*(*k*, *j*) for all *i* < *k* < *j*. The dashed line between two nucleotides means that the two nucleotides may or may not form a base pair. The solid line between two nucleotides means that the two nucleotides form a base pair.

That is, the normalized energy *NE*(*i*, *j*) is computed by taking the minimum of the following four cases:

- the total normalized energy of all loops in the optimal secondary structure *R*[*i*, *j* − 1] of subsequence *S* [*i*, *j* − 1] (Fig. 3a);
- the total normalized energy of all loops in the optimal secondary structure *R*[*i* + 1, *j*] of subsequence *S* [*i* + 1, *j*] (Fig. 3b);
- the total normalized energy of all loops in the optimal secondary structure *R*[*i*, *j*] of subsequence *S* [*i*, *j*], where *S* [*i*] and *S* [*j*] form a base pair (Fig. 3c);
- the minimum of *NE*(*i*, *k* – 1) plus *NE*(*k*, *j*) for all *i* < *k* < *j* (Fig. 3d).

Note that case (iii) of Equation (6) is not considered when the nucleotides at positions *i*, *j* are forbidden to form a base pair, i.e. (*S* [*i*], *S* [*j*]) is a non-standard base pair. A standard base pair is any of the following: (A, U), (U, A), (G, C), (C, G), (G, U), (U, G); all other base pairs are nonstandard.

#### Complexity

In calculating the time complexity of the folding algorithm, there is a need to check for finding the optimal *i*′, *j*′, *i* < *i*′ < *j*′ < *j*, in case (iii) (the optimal $i_{1}^{\prime },j_{1}^{\prime },i_{2}^{\prime },j_{2}^{\prime },\ldots ,i_{k}^{\prime },j_{k}^{\prime }$, $i<i_{1}^{\prime }<j_{1}^{\prime }<i_{2}^{\prime }<j_{2}^{\prime }<\ldots <i_{k}^{\prime }<j_{k}^{\prime }<j$, in case (iv), respectively) of Equation (5). Letting *n* be the number of nucleotides in the given sequence *S*, this checking would require *O*(*n*^4^) time in case (iii) and *O*(*n*^(*k*+ 2)^) time in case (iv). This time complexity can be reduced by adopting the following implementation strategy.

We introduce two entries to be updated based on the cases in Equation (6):

- *U*(*i*, *j*), which is the number of unpaired bases adjacent to *S* [*i*], *S* [*j*], *i* < *j*, where *S* [*i*], *S* [*j*] may or may not form a base pair;
- *H*(*i*, *j*), which is the set of exterior pairs (*p*, *q*), *i* < *p* < *q* < *j*, in the optimal secondary structure of subsequence *S* [*i*, *j*] if *S* [*i*], *S* [*j*] do not form a base pair, or simply the set of (*i, j*) if *S* [*i*]*, S* [*j*] form a base pair (recall that an interior pair of some loop is the exterior pair of another loop).

Depending on which case in Equation (6) yields the value of *NE*(*i*, *j*), we have:

(7) $$H(i,j)=\left\{\begin{array}{ll} H(i,j-1) & \text{if}\;\text{case}\;\text{(i)}\;\text{yields}\;\text{the}\;\text{value}\;\text{of}\;NE(i,j)\\ H(i+1,j) & \text{if}\;\text{case}\;(\text{ii})\;\text{yields}\;\text{the}\;\text{value}\;\text{of}\;NE(i,j)\\ \left\{(i,j)\right\} & \text{if}\;\text{case}\;(\text{iii})\;\text{yields}\;\text{the}\;\text{value}\;\text{of}\;NE(i,j)\\ H(i,h-1)\cup H(h,j),i<h<j & \text{if}\;\text{case}\;(\text{iv})\;\text{yields}\;\text{the}\;\text{value}\;\text{of}\;NE(i,j) \end{array}\right\}$$

Then:

(8) $$U(i,j)=\left\{\begin{array}{ll} U(i,j-1)+1 & \text{if}\;H(i,j)=H(i,j-1)\\ U(i+1,j)+1 & \text{if}\;H(i,j)=H(i+1,j)\\ 0 & \text{if}\;H(i,j)=\{(i,j)\}\\ U(i,h-1)+U(h,j) & \text{if}\;H(i,j)=H(i,h-1)\cup H(h,j),i<h<j \end{array}\right\}$$

With the two introduced entries, the case (iii) in Equation (5):

$$\underset{i<i^{\prime }<j^{\prime }<j}{\text{min}}\{E_{B}(i,j,i^{\prime },j^{\prime })+NE_{P}(i^{\prime },j^{\prime })\}$$

and the case (iv) in Equation (5):

$$\begin{array}{l} \underset{i<i_{1}^{\prime }<j_{1}^{\prime }<i_{2}^{\prime }<j_{2}^{\prime }<\ldots <i_{k}^{\prime }<j_{k}^{\prime }<j}{\text{min}}\{E_{J}(i,j,i_{1}^{\prime },j_{1}^{\prime },i_{2}^{\prime },j_{2}^{\prime },\ldots ,i_{k}^{\prime },j_{k}^{\prime })\\ \;\;\;\;\;\;\;\;\;\;\;\;\;\;\;\;\;\;\;\;\;\;\;\;\;\;\;\;\;\;\;\;\;\;\;\;\;+\sum_{r=1}^{k}NE_{P}(i_{r}^{\prime },j_{r}^{\prime })\} \end{array}$$

can be combined and expressed by a single formula as follows:

$$\underset{i<h<j}{\text{min}}\left\{\begin{array}{ll} \frac{e_{B}(i,j,i^{\prime },j^{\prime })}{\left(i^{\prime }-i+j-j^{\prime }+2\right)}+NE_{P}(i^{\prime },j^{\prime }) & \text{if}\;Z=\{(i^{\prime },j^{\prime })\}\\ \frac{e_{j}(i,j,i_{1}^{\prime },j_{1}^{\prime },i_{2}^{\prime },j_{2}^{\prime },\ldots ,i_{k}^{\prime },j_{k}^{\prime })}{U(i+1,h-1)+U(h,j-1)+2\times (|H(i+1,h-1)|+|H(h,j-1)|+1)} & \text{if}\;Z=\{(i_{1}^{\prime },j_{1}^{\prime }),(i_{2}^{\prime },j_{2}^{\prime }),\ldots ,(i_{k}^{\prime },j_{k}^{\prime })\}\\ +\sum_{r=1}^{k}NE_{P}(i_{r}^{\prime },j_{r}^{\prime }) & \end{array}\right\}$$

where *Z* = *H*(*i* + 1, *h* − 1) ∪ *H*(*h*, *j* − 1). Thus, it takes linear time to find the optimal *h*, *i* < *h* < *j*, that minimizes the above formula. Once the optimal *h* value is found, it takes constant time to find the optimal *i*′, *j*′ if *H*(*i* + 1, *h* − 1) ∪ *H*(*h*, *j* −1) = {(*i*′, *j*′)}, or the optimal $i_{1}^{\prime },j_{1}^{\prime },i_{2}^{\prime },j_{2}^{\prime },\ldots ,i_{k}^{\prime },j_{k}^{\prime }$ if *H*(*i* + 1, *h* − 1) ∪ *H*(*h*, *j* − 1) = {( $i_{1}^{\prime },j_{1}^{\prime }$), ( $i_{2}^{\prime },j_{2}^{\prime }$), ..., ( $i_{k}^{\prime },j_{k}^{\prime }$)}. Therefore, it takes linear time to compute *NE_P_* (*i*, *j*) in Equation (5). Hence, the time complexity of the folding algorithm is *O*(*n*^3^) since we need to calculate *NE_P_* (*i*, *j)* for all 1 ≤ *i* < *j* ≤ *n*, where *n* is the number of nucleotides in the given sequence *S*. The space complexity of the folding algorithm is *O*(*n*^2^), since all energy values are stored in two dimensional tables. The normalized energy of the optimal secondary structure *R* for the sequence *S* equals *NE*(1, *n*).

### Calculation of covariance scores

When applying the above folding algorithm to a multiple sequence alignment *A*_o_, we take into consideration the correlation between columns of the alignment. In many cases, the sequences in the alignment may have highly varying lengths. We refine the alignment *A*_o_ by deleting columns containing more than 75% gaps to get a refined alignment *A*.^17^ We will use this refined alignment throughout the rest of this subsection.

#### Covariance score

We use the covariance score introduced by RNAa-lifold^15^ to quantify the relationship between two columns in the refined alignment. Let *f_ij_*(*XY*) be the frequency of finding both base *X* in column *i* and base *Y* in column *j*, where *X*, *Y* are in the same row of the refined alignment. We exclude the occurrences of gaps in column *i* or column *j* when calculating *f_ij_*(*XY*). The covariation measure for columns *i*, *j*, denoted *C_ij_*, is calculated by Equation (9):

(9) $$C_{ij}=\frac{\sum_{XY,X^{\prime }Y^{\prime }}f_{ij}(XY)D_{ij}(XY,X^{\prime }Y^{\prime })f_{ij}(X^{\prime }Y^{\prime })}{2}$$

Here, *D_ij_*(*XY*, *X′Y′*) is the Hamming distance between the two base pairs (*X*, *Y*) and (*X*′, *Y*′) if both of the base pairs are standard base pairs, or 0 otherwise. The Hamming distance between (*X*, *Y*) and (*X*′, *Y*′) is calculated as follows:

(10) $$D_{ij}(XY,X^{\prime }Y^{\prime })=2-\delta (X,X^{\prime })-\delta (Y,Y^{\prime })$$

where

(11) $$\delta (X,X^{\prime })=\{\begin{array}{cc} 1 & \text{if}\;X=X^{\prime },\\ 0 & \text{otherwise}\text{.} \end{array}$$

Observe that the information acquired from the two base pairs (*X*, *Y*) and (*X*′, *Y*′) is the same as that from (*X*′, *Y*′) and (*X*, *Y*). Thus, we divide the numerator in Equation (9) by 2 so as to obtain the non-redundant mutual information between column *i* and column *j* in the refined alignment.

For every pair of columns *i*, *j* in the refined alignment, the covariance score of the two columns *i* and *j*, denoted *Cov_ij_*, is calculated in Equation (12):

(12) $$Cov_{ij}=C_{ij}+c_{1}\times NF_{ij}$$

Here, *C_ij_* is as defined in Equation (9)*c*_1_ is a user-defined coefficient (in the study presented here, *c*_1_ has a value of −1), and

(13) $$NF_{ij}=\frac{NC_{ij}}{N}$$

where *N* is the total number of sequences and *NC_ij_* is the total number of conflicting sequences in the refined alignment. A conflicting sequence is one that has a gap in column *i* or column *j*, or has a non-standard base pair in the columns *i*, *j* of the refined alignment. A sequence with gaps in both columns *i*, *j* is not conflicting.

#### Pairing threshold

We say that column *i* and column *j* in the refined alignment can possibly form a base pair if their covariance score is greater than or equal to a pairing threshold; otherwise, column *i* and column *j* are forbidden to form a base pair. The pairing threshold, *η*, used in RSpredict is calculated as follows.

It is known that, on average, 54% of the nucleotides in an RNA sequence *S* are involved in the base pairs of its secondary structure.^22^ We use this information to calculate an alignment-dependent pairing threshold, observing that the base pairs in the consensus secondary structure of a sequence alignment represent the column pairs with the highest covariance scores. Given that different structures contain different numbers of base pairs, we consider two different percentages of columns, namely, 30% and 65%, in the sequence alignment. For each percentage *p*, there are at most *T_p_* possible base pairs, where

(14) $$T_{P}=\frac{\left(p\times n)\times (p\times n-1\right)}{2}$$

and *n* is the number of columns in the sequence alignment.

**Figure 4.**
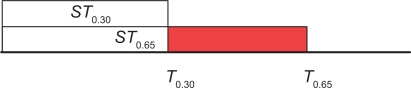
Illustration of the pairing threshold computation. The pairing threshold used in RSpredict is computed as the average of the covariance scores inside the red shaded area.

Now, we calculate the covariance scores of all pairs of columns in the given refined alignment, and sort the covariance scores in descending order. We then select the top *T_p_* largest covariance scores and store the covariance scores in the set *ST_p_*. Thus, the set *ST*_0.65_ contains the top largest covariance scores that involve 65% of the columns in the refined alignment; the set *ST*_0.30_ contains the top largest covariance scores that involve 30% of the columns in the refined alignment; and *ST*_0.65_\*ST*_0.30_ is the set difference that contains covariance scores in *ST*_0.65_ but not in *ST*_0.30_ (see Fig. 4). The pairing threshold *η* used in RSpredict is calculated as the average of the covariance scores in *ST*_0.65_*\ST*_0.30_, as shown in Equation (15):

(15) $$\eta =\frac{\sum_{Cov_{ij}\in (ST_{0.65}\backslash ST_{0.30})}Cov_{ij}}{\left|ST_{0.65}\backslash ST_{0.30}\right|}$$

where the denominator is the cardinality of the set difference *ST*_0. 65_\*ST*_0.30_.

If the covariance score of columns *i* and *j* is greater than or equal to *η*, then column *i* and column *j* can possibly form a base pair, and we refer to (*i*, *j*) as a pairing column. If the covariance score of the columns *i* and *j* is less than *η*, we will check the covariance scores of the immediate neighboring column pairs of *i*, *j* to see if they are above a user-defined threshold^20^ (in the study presented here, this threshold is set to 0). The immediate neighboring column pairs of *i*, *j* are *i* + 1, *j* −1 and *i* − 1, *j* + 1. If the covariance scores of both of the immediate neighboring column pairs of *i*, *j* are greater than or equal to *max*{*η*, 0}, then (*i*, *j*) is still considered as a paring column.

### Algorithms for RSpredict

Given a refined multiple sequence alignment *A* with *N* sequences, let (*i*, *j*) be a pairing column in *A*. Let $X_{i}^{S}$ ( $Y_{j}^{S}$, respectively) be the nucleotide at position *i* (*j*, respectively) of the sequence *S* in the alignment *A*. ( $X_{i}^{S},Y_{j}^{S}$) must be the exterior pair of some loop *L* in *S*. We use *e*( $X_{i}^{S},Y_{j}^{S}$) to represent the free energy of that loop *L*. If ( $X_{i}^{S},Y_{j}^{S}$) is a nonstandard base pair, *e*( $X_{i}^{S},Y_{j}^{S}$) = 0. We assign the pairing column (*i*, *j*) a pseudo-energy value *e_ij_* where

(16) $$e_{ij}=\frac{1}{N}\sum_{s\in A}e(X_{i}^{s},Y_{j}^{s})+c_{2}\times Cov_{ij}$$

Here, *c*_2_ is a user-dedined coefficient (in the study presented here, *c*_2_ = −1). Thus, every pairing column in the refined alignment *A* has a pseudo-energy value. We then apply the minimum normalized energy folding algorithm described in the beginning of this section to the refined alignment *A*, treating each pairing column in *A* as a possible base pair considered in the folding algorithm.

Notice that when calculating the normalized energy for the loop *L*, the sequence *S* is in the refined alignment *A*, which may have fewer columns than the original input alignment *A*_o_ (cf. Fig. 2). RSpredict computes all normalized energies based on the refined alignment, and the program uses loop lengths from the refined alignment *A* rather than the original input alignment *A*_o_. Let *R* be the consensus secondary structure, computed by RSpredict, for the refined alignment *A*. We obtain the consensus structure *R*_o_ of the original input alignment *A*_o_ by inserting unpaired gaps to the positions in *R* whose corresponding columns are deleted when getting *A* from *A*_o_ (cf. Fig. 2). The following summarizes the algorithms for RSpredict:

1. Input an alignment *A*_o_ in FASTA or ClustalW format.
2. Delete the columns with more than 75% gaps from *A*_o_ to obtain a refined alignment *A*.
3. Compute the pseudo-energy *e_ij_* for every pairing column (*i*, *j*) in *A* as in Equation (16).
4. Run the minimum normalized energy folding algorithm on *A*, using the pseudo-energy values obtained from Step (3) to produce the consensus secondary structure *R* of the refined alignment *A*. The base at position *i* of the consensus secondary structure *R* is the most frequently occurring nucleotide, excluding gaps, in the *i*th column of the refined alignment *A*.
5. Map the consensus structure *R* back to the original alignment *A*_o_ by inserting unpaired gaps to the positions of *R* whose corresponding columns are deleted in Step (2).

Notice that Equation (6) is used to compute the *NE* values only. To generate the optimal structure *R* in Step (4), we maintain a stack of pointers that point to the substructures of loops with minimum normalized energy as we compute the *NE* values. Once all the *NE* values are calculated and the normalized energy of the optimal secondary structure *R* is obtained, we pop up the pointers from the stack to extract the optimal predicted structure. In Step (5), we map the bases (base pairs, respectively) for the columns (column pairs, respectively) in *A* to their corresponding columns (column pairs, respectively) in *A*_o_. For example, consider Figure 2 again. In the figure, the refined alignment *A* is obtained by deleting column 4 from the original input alignment *A*_o_. The bases for columns 1, 2, 3, 4 in *A* are mapped to columns 1, 2, 3, 5 in *A*_o_. The base pair between column 1 and column 9 in *A* becomes the base pair between column 1 and column 10 in *A*_o_; the base pair between column 2 and column 8 in *A* becomes the base pair between column 2 and column 9 in *A*_o_. An unpaired gap is inserted to the position corresponding to the deleted column 4 in *A*_o_.

Let *N* be the number of sequences and *n*_o_ be the number of columns in the input alignment *A*_o_. Step (2) takes *O*(*Nn*_o_) time and space. Step (3) takes $O(n_{\text{o}}^{2})$ time and space. Step (4) takes $O(n_{\text{o}}^{3})$ time and $O(n_{\text{o}}^{2})$ space. Step (5) takes *O*(*n*_o_) time and space. Therefore, the time complexity of RSpredict is $O(Nn_{\text{o}}+n_{\text{o}}^{3})$, which is approximately $O(n_{\text{o}}^{3})$ as *N* is usually much smaller than *n*_o_, and the space complexity of RSpredict is $O(Nn_{\text{o}}+n_{\text{o}}^{2})$, which is approximately $O(n_{\text{o}}^{2})$.

## Results

We conducted a series of experiments to evaluate the performance of RSpredict and compared it with three related tools including KNetFold, Pfold and RNAalifold. We tested these tools on Rfam^23^ sequence alignments with different similarities. The Rfam sequence alignments come with consensus structures. For evaluation purposes, we used the Rfam consensus structures as reference structures and compared them against the consensus structures predicted by the four tools. The similarity of a sequence alignment is determined by the average pairwise sequence identity (APSI) of that alignment. In the study presented here, a sequence alignment is of high similarity if its APSI value is greater than 75%, is of medium similarity if its APSI value is between 55% and 75%, or is of low similarity if its APSI value is less than 55%. The data sets used in testing included 20 Rfam sequence alignments of high similarity and 36 Rfam sequence alignments of low and medium similarity. These data sets were chosen to form a collection of sequence alignments with different (low, medium and high) APSI values, different numbers of sequences, as well as different sequence alignment lengths. More specifically, the data sets contained sequence alignments that ranged in size from 2 to 160 sequences, in length from 33 to 262 nucleotides and had APSI values ranging from 42% to 99%. We also tested the tools on a multiple sequence alignment obtained from our study on the *Drosophila* X chromosome.^24^,^25^ The *Drosophila* data set has a reference structure, obtained from biochemical and other methods that are different from the algorithms used in the tools under analysis.

The performance measures used in our study include sensitivity (SN) and selectivity (SL),^20^ where

(17) $$SN=\frac{TP}{TP+FN}$$

(18) $$SL=\frac{TP}{TP+(FP-\xi )}$$

Here *TP* is the number of correctly predicted base pairs (“true positives”), *FN* is the number of base pairs in a reference structure that were not predicted (“false negatives”) and *FP* is the number of incorrectly predicted base pairs (“false positives”). False positives are classified as inconsistent, contradicting or compatible.^20^ In predicting the consensus secondary structure for a multiple sequence alignment, a predicted base pair (*i*, *j*) is inconsistent if column *i* in the alignment is paired with column *q*, *q ≠ j*, or column *j* is paired with column *p*, *p ≠ i*, and *p*, *q* form a base pair in the reference structure of the alignment. A base pair (*i*, *j*) is contradicting if there exists a base pair (*p*, *q*) in the reference structure of the alignment, such that *i* < *p* < *j* < *q* or *p* < *i* < *q* < *j*. A base pair (*i*, *j*) is compatible if it is a false positive but is neither inconsistent nor contradicting. The *ξ* in *SL* represents the number of compatible base pairs, which are considered neutral with respect to algorithmic accuracy. Therefore *ξ* is subtracted from *FP*. Finally, we used the Matthews correlation coefficient (MCC) to combine the sensitivity and selectivity, where MCC is approximated to the geometric mean of the two measures, i.e. $MCC\approx \sqrt{SN\times SL}$. The larger MCC, SN, SL values a tool has, the better performance that tool achieves and the more accurate that tool is.

### Performance evaluation on Rfam alignments of high similarity

The first data set consisted of seed alignments of high similarity taken from 20 families in Rfam. The APSI values of these seed alignments ranged from 77% to 99%. The alignments ranged in size from 2 to 160 sequences and in length from 33 to 159 nucleotides. Table 1 presents details concerning the 20 families and their seed alignments.

**Figure 5.**
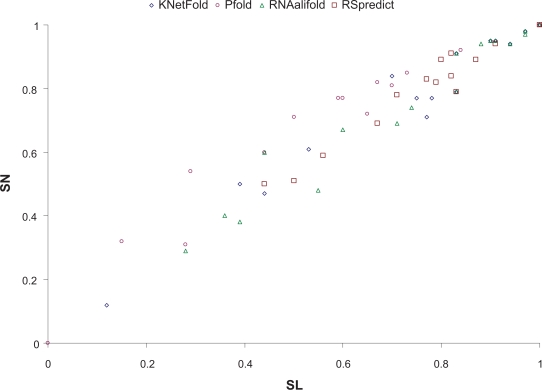
Comparison of the SN and SL values of the four tools under analysis on the seed alignments of high similarity taken from the 20 families listed in Table 1. We use ROC plots to simultaneously display the sensitivity and selectivity values achieved by each tool. The X-axis shows the selectivity values, whereas the Y-axis shows the sensitivity values.

All four tools including RSpredict, KNetFold, RNAalifold and Pfold were tested on this data set. Table 2 presents the values of Matthews correlation coefficient (MCC), sensitivity (SN) and selectivity (SL), as well as their mean and standard deviation for each of the four tools. Figure 5 compares the four tools based on these values. We use ROC (receiver operating characteristic) plots to simultaneously display both sensitivity and selectivity for each tool.

It can be seen from Table 2 and Figure 5 that RSpredict performed the best. The Pfold tool had good performance in selectivity but did not perform well in sensitivity and as a result in Matthews correlation coefficient. It also suffered from a size limitation (the Pfold web server can accept a multiple alignment of up to 40 sequences). Only 17 out of the 20 sequence alignments used in the experiment were accepted by the Pfold server; the other three alignments (RF00386, RF00041 and RF00389) had more than 40 sequences and therefore could not be run on the Pfold server. RSpredict had stable performance with the best mean 0.85 (standard deviation 0.16, respectively) in MCC, while the other methods’ MCC values varied a lot and had means (standard deviations, respectively) ranging from 0.71 to 0.82 (0.24 to 0.28, respectively).

### Performance evaluation on Rfam alignments of medium and low similarity

In the second experiment, we compared RSpredict with the other three methods on multiple sequence alignments of low and medium similarity. The test dataset included seed alignments of 36 families taken from Rfam.^23^ The average pairwise sequence identity (APSI) values of the seed alignments ranged from 42% to 75%, the number of sequences in the alignments ranged from 3 to 114, and the alignment lengths ranged from 43 to 262 nucleotides. Table 3 presents details concerning the 36 families and their seed alignments.

**Figure 6.**
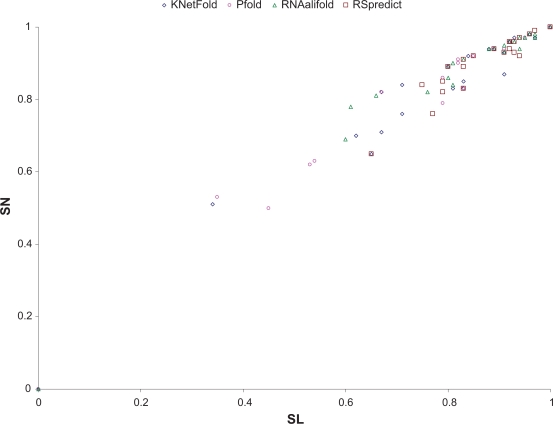
Comparison of the SN and SL values of the four tools under analysis on the seed alignments of low and medium similarity taken from the 36 families listed in Table 3. We use ROC plots to simultaneously display the sensitivity and selectivity values achieved by each tool. The X-axis shows the selectivity values, whereas the Y-axis shows the sensitivity values.

Table 4 presents the values of Matthews correlation coefficient (MCC), sensitivity (SN) and selectivity (SL), respectively, as well as their mean and standard deviation for each of the four tools. Figure 6 shows ROC (receiver operating characteristic) plots, which simultaneously display both sensitivity and selectivity for each tool.

Comparing Table 2 and Table 4, we see that the methods under analysis generally performed better on sequence alignments of medium and low similarity than on sequence alignments of high similarity. RSpredict outperformed the other three methods based on the three performance measures used in the experiment. The tool achieved a high mean value of 0.94 in MCC, better than those of KNetFold (0.86), Pfold (0.88) and RNAalifold (0.89). Similar results were observed for sensitivity and selectivity values. Furthermore, RSpredict exhibited stable performance across all the families tested in the experiment. The tool had an MCC, SN and SL standard deviation of 0.08, 0.09 and 0.08, respectively. These numbers were better than the standard deviation values obtained from the other three methods, which ranged from 0.12 to 0.25. Due to the restriction on the alignment size imposed by the Pfold server, only 27 alignments out of 36 could be run on Pfold. The other nine alignments had more than 40 sequences and hence could not be accepted by the Pfold server.

### Performance evaluation on the *Drosophila* dataset

**Figure 7.**
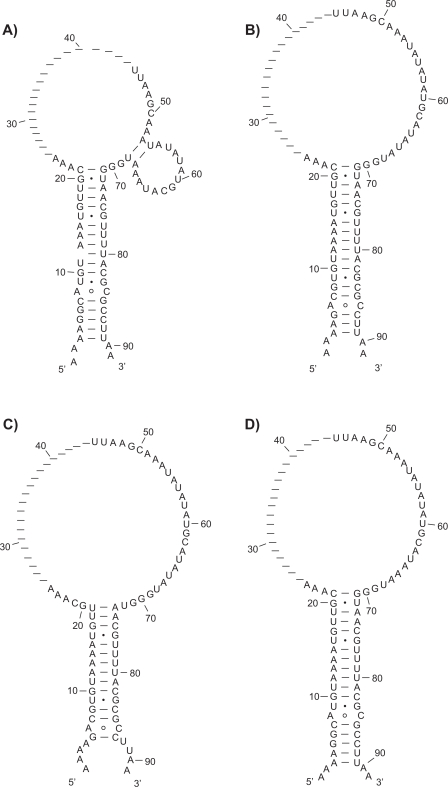
Consensus structures generated from the roX2 RNA sequence alignment. **A**) The consensus structure produced by RSpredict. **B**) The consensus structure predicted by KNetFold. **C**) The consensus structure predicted by Pfold. **D**) The consensus structure predicted by RNAalifold. RSpredict, KNetFold and RNAalifold, all with MCC = 1, performed better than Pfold with MCC value of 0.88. Note that each gap in the consensus structure in (a) represents a column with more than 75% gaps in the roX2 RNA sequence alignment. All the columns with more than 75% gaps are deleted to obtain the refined alignment based on which RSpredict generates the consensus structure.

The male-specific lethal (MSL) complex, which includes two noncoding RNAs on X (*roX*1 and *roX* 2 RNAs), induces histone H4-Lys16 acetylation for twofold hypertranscription of the male X chromosome in *Drosophila melanogaster*.^24^,^25^ We applied all four methods including RSpredict, KNetFold, Pfold and RNAalifold to predicting a common secondary structure of *roX* 2 RNA. Among the sequences of the *roX* 2 gene (1.4 kb) found from nine different *Drosophila* species, an evolutionarily conserved functional domain (71 nt) was characterized and predicted as a stem-loop structure by RSpredict, KNetFold and RNAalifold respectively (Figs. 7a, 7b and 7d respectively). This result affirmed what our lab experiments revealed.^24^ Pfold predicted a less accurate structure with a shorter stem (Fig. 7c).

## Discussion

In this paper we presented a software tool, called RSpredict, capable of predicting the consensus secondary structure for a set of aligned RNA sequences via pseudo-energy minimization. Our experimental results showed that RSpredict is competitive with some widely used tools including RNAali-fold and Pfold on tested datasets, suggesting that RSpredict can be a choice when biologists need to predict RNA secondary structures of multiple sequence alignments. Notice that RSpredict differs from our previously developed KNetFold^20^ in that KNetFold is a machine learning method that relies on pre-compiled training data derived from existing RNA secondary structures. RSpredict, on the other hand, is based on a dynamic programming algorithm for folding sequences and does not utilize training data.

RSpredict adopts the normalized energy concept originated from Densityfold, whose web server is named alteRNA.^5^ We described the difference between RSpredict and Densityfold in the beginning of the Introduction section. Unlike RSpredict that predicts consensus structures for multiple sequence alignments, alteRNA predicts secondary structures for single sequences. Thus, one cannot do a direct comparison of performance between the two tools. We implemented the idea in alteRNA in a program that uses the same covariance scores as RSpredict to predict consensus structures for multiple sequence alignments. The performance of this program is inferior to those of the four tools studied in the paper.

RSpredict contains two user-defined parameters, *c*_1_ in Equation (12) and *c*_2_ in Equation (16). In the study presented here, both *c*_1_ and *c*_2_ were fixed at −1. Changing the value of *c*_1_ or *c*_2_ would affect the accuracy of the predicted structure. More specifically, when *c*_1_ is smaller than −1, the accuracy degrades slowly. When *c*_1_ is set to 0, the predicted consensus structures are less accurate than when *c*_1_ is set to −1. This shows the impact that conflicting sequences in an alignment have on consensus structure prediction, since when *c*_1_ is 0, there is no weight for the conflicting sequences in covariance score calculation. When *c*_1_ is set to a positive number, RSpredict generates less accurate consensus structures. The same behavior was observed for *c*_2_. In practice, if a few more base pairs are predicted or omitted, given that all the others are in the right places, the predicted structure would not be optimal, i.e. its pseudo-energy would not be minimized. A tool predicting fewer base pairs would have a lower sensitivity value whereas a tool predicting more base pairs might have a lower selectivity value.

It was observed that the accuracy of the consensus structures produced by RSpredict varies with sequence lengths, though there is no clear trend between the accuracy and the lengths. For example, referring to Tables 3 and 4, the seed alignment of RF00230 (RF00559 and RF00468, respectively) has a length of 262 nt (81 nt and 66 nt, respectively) and MCC value of 0.83 (0.91 and 0.65, respectively), showing no direct relationship between the alignment length and the MCC.

Finally, we compared the computational time required by the four tools studied in the paper. Pfold and KNetFold are available as web servers. We submitted the same multiple alignments having a length of 100 nt, 200 nt and 300 nt, respectively to each of the four web servers. Results were reported back by KNetFold (Pfold, RNAalifold, RSpredict, respectively) in 605, 660, 1125 seconds (10, 15, 33 seconds, 9, 10, 11 seconds, 9, 19, 76 seconds, respectively). While the speed of a web server also depends on network traffic and server workload, these timing data give an estimate of how fast each tool can run. The RSpredict program needs 0.3 MB RAM (1.3 MB and 2.9 MB, respectively) to perform the computation for the input alignment whose length is 100 nt (200 nt and 300 nt, respectively) measured on a PC with Intel(R) Core(TM)2 Duo CPU (2.19 GHz/2.00 GB RAM/Microsoft Windows XP). The RSpredict web server can accept multiple alignments with at most 200 sequences in size and at most 500 nucleotides in length, though the downloadable version does not have this restriction.

All the four tools studied in this paper take multiple sequence alignments as input data. We are looking into algorithms for enhancing the quality of multiple sequence alignments so as to improve on secondary structure prediction. One approach is to take into consideration the effect of base-pair covariation in the alignment process, like the Murlet tool.^26^ There are other approaches for obtaining better sequence alignments. For example, BlockMSA^27^ uses a combination of a biclustering algorithm and a divide-and-conquer technique. Groups of similar sequences are found and subsequences within them are locally aligned. The final alignment is obtained by dividing both the set of sequences and their contents.^27^ Other ways to improve the prediction accuracy include the utilization of evolutionary information, more sophisticated models of covariance scoring, and training data for more accurate pairing thresholds. Finally, by integrating our previously developed RADAR server^6^ with RSpredict, we plan to offer our software as a suite of tools to the user as done in some recent work.^28^

## Figures and Tables

**Table 1.** The accession number, description, number of sequences, and length of the seed alignment of each of the 20 families used in the experiment.

**Accession**	**Description**	**Number of seqs**	**Length**	**APSI**
RF00460	U1A polyadenylation inhibition element (PIE)	8	75	77%
RF00326	Small nucleolar RNA Z155	8	81	79%
RF00560	Small nucleolar RNA SNORA17	38	132	82%
RF00453	Cardiovirus cis-acting replication element (CRE)	12	33	82%
RF00386	Enterovirus 5’ cloverleaf cis-acting replication element	160	91	83%
RF00421	Small nucleolar RNA SNORA32	9	122	84%
RF00302	Small nucleolar RNA SNORA65	8	130	84%
RF00465	Japanese encephalitis virus (JEV) hairpin structure	20	60	86%
RF00501	Rotavirus cis-acting replication element (CRE)	14	68	87%
RF00041	Enteroviral 3′ UTR element	60	123	87%
RF00575	Small nucleolar RNA SNORD70	4	88	89%
RF00362	Pospiviroid RY motif stem loop	16	79	92%
RF00105	Small nucleolar RNA SNORD115	23	82	92%
RF00467	Rous sarcoma virus (RSV) primer binding site (PBS)	23	75	93%
RF00389	Bamboo mosaic virus satellite RNA cis-regulatory element	42	159	93%
RF00384	Poxvirus AX element late mRNA cis-regulatory element	7	62	93%
RF00098	Snake H/ACA box small nucleolar RNA	22	150	93%
RF00607	Small Nucleolar RNA SNORD98	2	67	98%
RF00320	Small nucleolar RNA Z185	2	86	98%
RF00318	Small nucleolar RNA Z175	3	81	99%

The seed alignments of the 20 families from Rfam are of high similarity. Their average pairwise sequence identity (APSI) values are shown in the last column of the table.

**Table 2.** The MCC, SN and SL values for each method tested in the experiment with sequence alignments of high similarity, as well as the mean and standard deviation of the MCC, SN and SL values for each tool under analysis.

	**KNetFold**			**Pfold**			**RNAalifold**			**RSpredict**		
**Accession**	**MCC**	**SN**	**SL**	**MCC**	**SN**	**SL**	**MCC**	**SN**	**SL**	**MCC**	**SN**	**SL**
RF00460	**1.00**	1.00	1.00	**1.00**	1.00	1.00	0.69	0.71	0.67	**1.00**	1.00	1.00
RF00326	**1.00**	1.00	1.00	0.71	0.50	1.00	0.91	0.83	1.00	**1.00**	1.00	1.00
RF00560	**0.95**	0.91	1.00	0.00	0.00	0.00	**0.95**	0.91	1.00	0.83	0.77	0.89
RF00453	0.84	0.70	1.00	0.77	0.60	1.00	0.67	0.60	0.75	**0.89**	0.80	1.00
RF00386	**1.00**	1.00	1.00	N/A	N/A	N/A	0.97	0.97	0.97	**1.00**	1.00	1.00
RF00421	0.79	0.83	0.76	**0.81**	0.70	0.94	0.79	0.83	0.76	0.79	0.83	0.76
RF00302	0.77	0.75	0.80	0.92	0.84	1.00	**0.98**	0.97	1.00	0.94	0.91	0.97
RF00465	0.50	0.39	0.64	0.31	0.28	0.36	0.29	0.28	0.31	**0.59**	0.56	0.63
RF00501	0.12	0.12	0.12	0.54	0.29	1.00	**0.94**	0.88	1.00	0.78	0.71	0.86
RF00041	**0.98**	0.97	1.00	N/A	N/A	N/A	0.95	0.90	1.00	0.82	0.79	0.85
RF00575	0.71	0.77	0.65	**0.85**	0.73	1.00	0.48	0.55	0.41	0.84	0.82	0.86
RF00362	0.77	0.78	0.75	0.72	0.65	0.79	0.74	0.74	0.74	**0.89**	0.87	0.91
RF00105	0.47	0.44	0.50	**0.60**	0.44	0.80	**0.60**	0.44	0.80	0.50	0.44	0.57
RF00467	**1.00**	1.00	1.00	**1.00**	1.00	1.00	**1.00**	1.00	1.00	**1.00**	1.00	1.00
RF00389	**0.61**	0.53	0.71	N/A	N/A	N/A	0.38	0.39	0.36	0.51	0.50	0.51
RF00384	**0.94**	0.94	0.94	0.77	0.59	1.00	**0.94**	0.94	0.94	0.91	0.82	1.00
RF00098	**0.95**	0.90	1.00	0.32	0.15	0.67	0.40	0.36	0.45	0.69	0.67	0.72
RF00607	**1.00**	1.00	1.00	**1.00**	1.00	1.00	**1.00**	1.00	1.00	**1.00**	1.00	1.00
RF00320	0.91	0.83	1.00	0.82	0.67	1.00	0.91	0.83	1.00	**1.00**	1.00	1.00
RF00318	**1.00**	1.00	1.00	**1.00**	1.00	1.00	**1.00**	1.00	1.00	**1.00**	1.00	1.00

Mean	0.82	0.79	0.84	0.71	0.61	0.86	0.78	0.76	0.81	**0.85**	0.82	0.88
Std. Dev.	0.24	0.25	0.23	0.28	0.31	0.28	0.24	0.24	0.24	**0.16**	0.18	0.16

The best MCC value for each family, and the best mean and standard deviation are highlighted in bold. Note that only 17 out of the 20 sequence alignments in this experiment were accepted as input to the Pfold server. The other three (RF00386, RF00041 and RF00389) had more than 40 sequences and therefore could not be run on the server. Note also that RSpredict has the best standard deviation in MCC, SN and SL respectively compared with the other three tools. This shows that RSpredict has stable performance across all the families tested in the experiment.

**Table 3.** The accession number, description, number of sequences, and length of the seed alignment of each of the 36 families used in the experiment.

**Accession**	**Description**	**Number of seqs**	**Length**	**APSI**
RF00230	T-box leader	103	262	42%
RF00080	yybP-ykoY leader	50	131	44%
RF00515	PyrR binding site	72	125	47%
RF00557	Ribosomal protein L10 leader	66	149	48%
RF00504	Glycine riboswitch	93	111	50%
RF00029	Group II catalytic intron	114	94	52%
RF00458	Cripavirus internal ribosome entry site (IRES)	7	203	54%
RF00559	Ribosomal protein L21 leader	33	81	54%
RF00234	glmS glucosamine-6-phosphate activated ribozyme	11	218	55%
RF00556	Ribosomal protein L19 leader	24	43	55%
RF00519	suhB	13	80	56%
RF00379	ydaO/yuaA leader	25	150	58%
RF00380	ykoK leader	36	172	59%
RF00445	mir-399 microRNA precursor family	13	119	59%
RF00522	PreQ1 riboswitch	22	47	59%
RF00095	Pyrococcus C/D box small nucleolar RNA	25	59	60%
RF00442	ykkC-yxkD leader	11	111	60%
RF00430	Small nucleolar RNA SNORA54	5	134	60%
RF00521	SAM riboswitch (alpha-proteobacteria)	12	79	61%
RF00049	Small nucleolar RNA SNORD36	20	82	63%
RF00513	Tryptophan operon leader	11	100	63%
RF00309	Small nucleolar RNA snR60/Z15/Z230/Z193/J17	23	106	63%
RF00451	mir-395 microRNA precursor family	21	112	64%
RF00464	mir-92 microRNA precursor family	33	80	64%
RF00507	Coronavirus frameshifting stimulation element	23	85	66%
RF00388	Qa RNA	5	103	70%
RF00357	Small nucleolar RNA R44/J54/Z268 family	19	105	70%
RF00434	Luteovirus cap-independent translation element (BTE)	17	108	71%
RF00525	Flavivirus DB element	111	76	71%
RF00581	Small nucleolar SNORD12/SNORD106	8	91	71%
RF00238	ctRNA	48	88	72%
RF00477	Small nucleolar RNA snR66	5	105	72%
RF00608	Small Nucleolar RNA SNORD99	3	80	72%
RF00468	Hepatitis C virus stem-loop VII	110	66	74%
RF00489	ctRNA	14	80	74%
RF00113	QUAD RNA	14	150	75%

The seed alignments of the 36 families from Rfam are of low and medium similarity. Their average pairwise sequence identity (APSI) values are shown in the last column of the table.

**Table 4.** The MCC, SN and SL values for each method tested in the experiment with sequence alignments of low and medium similarity, as well as the mean and standard deviation of the MCC, SN and SL values for each tool under analysis.

	**KNetFold**			**Pfold**			**RNAalifold**			**RSpredict**		
**Accession**	**MCC**	**SN**	**SL**	**MCC**	**SN**	**SL**	**MCC**	**SN**	**SL**	**MCC**	**SN**	**SL**
RF00230	0.76	0.71	0.82	N/A	N/A	N/A	0.69	0.60	0.79	**0.83**	0.83	0.83
RF00080	0.87	0.91	0.83	N/A	N/A	N/A	0.90	0.81	1.00	**0.97**	0.94	1.00
RF00515	0.84	0.71	1.00	N/A	N/A	N/A	0.81	0.66	1.00	**0.96**	0.93	1.00
RF00557	**1.00**	1.00	1.00	N/A	N/A	N/A	0.97	0.94	1.00	0.97	0.94	1.00
RF00504	**0.93**	0.91	0.95	N/A	N/A	N/A	**0.93**	0.91	0.95	**0.93**	0.91	0.95
RF00029	**1.00**	1.00	1.00	N/A	N/A	N/A	**1.00**	1.00	1.00	**1.00**	1.00	1.00
RF00458	0.51	0.34	0.75	0.94	0.91	0.98	**0.97**	0.95	0.98	0.76	0.77	0.74
RF00559	0.71	0.67	0.75	**0.94**	0.89	1.00	0.78	0.61	1.00	0.91	0.83	1.00
RF00234	0.92	0.84	1.00	0.94	0.89	1.00	0.97	0.97	0.97	**1.00**	1.00	1.00
RF00556	**0.89**	0.80	1.00	**0.89**	0.80	1.00	**0.89**	0.80	1.00	**0.89**	0.80	1.00
RF00519	**1.00**	1.00	1.00	**1.00**	1.00	1.00	**1.00**	1.00	1.00	**1.00**	1.00	1.00
RF00379	**0.98**	0.96	1.00	0.96	0.92	1.00	**0.98**	0.96	1.00	0.94	0.92	0.96
RF00380	0.83	0.81	0.85	**0.86**	0.79	0.93	0.84	0.81	0.87	0.82	0.79	0.84
RF00445	**1.00**	1.00	1.00	0.94	0.89	1.00	0.96	0.93	1.00	**1.00**	1.00	1.00
RF00522	**1.00**	1.00	1.00	**1.00**	1.00	1.00	**1.00**	1.00	1.00	**1.00**	1.00	1.00
RF00095	0.82	0.67	1.00	0.82	0.67	1.00	0.00	0.00	0.00	**1.00**	1.00	1.00
RF00442	**1.00**	1.00	1.00	0.97	0.95	1.00	**1.00**	1.00	1.00	**1.00**	1.00	1.00
RF00430	0.97	0.93	1.00	**1.00**	1.00	1.00	0.98	0.97	1.00	0.93	0.93	0.93
RF00521	**1.00**	1.00	1.00	0.83	0.83	0.83	**1.00**	1.00	1.00	**1.00**	1.00	1.00
RF00049	**1.00**	1.00	1.00	**1.00**	1.00	1.00	**1.00**	1.00	1.00	**1.00**	1.00	1.00
RF00513	0.83	0.83	0.83	0.63	0.54	0.72	**0.91**	0.83	1.00	0.84	0.75	0.95
RF00309	**1.00**	1.00	1.00	**1.00**	1.00	1.00	**1.00**	1.00	1.00	**1.00**	1.00	1.00
RF00451	0.97	0.97	0.97	0.62	0.53	0.73	0.86	0.80	0.92	**1.00**	1.00	1.00
RF00464	**0.94**	0.88	1.00	0.53	0.35	0.82	**0.94**	0.88	1.00	0.92	0.85	1.00
RF00507	**1.00**	1.00	1.00	0.90	0.82	1.00	0.95	0.91	1.00	**1.00**	1.00	1.00
RF00388	**1.00**	1.00	1.00	0.91	0.82	1.00	**1.00**	1.00	1.00	0.99	0.97	1.00
RF00357	**1.00**	1.00	1.00	**1.00**	1.00	1.00	**1.00**	1.00	1.00	**1.00**	1.00	1.00
RF00434	0.70	0.62	0.78	0.50	0.45	0.57	0.82	0.76	0.88	**0.85**	0.79	0.92
RF00525	**0.94**	0.89	1.00	N/A	N/A	N/A	**0.94**	0.89	1.00	0.92	0.94	0.89
RF00581	0.94	0.89	1.00	0.94	0.89	1.00	**1.00**	1.00	1.00	0.94	0.89	1.00
RF00238	**1.00**	1.00	1.00	N/A	N/A	N/A	0.91	0.83	1.00	0.89	0.83	0.95
RF00477	0.00	0.00	0.00	**1.00**	1.00	1.00	0.00	0.00	0.00	**1.00**	1.00	1.00
RF00608	0.00	0.00	0.00	**1.00**	1.00	1.00	**1.00**	1.00	1.00	**1.00**	1.00	1.00
RF00468	0.65	0.65	0.65	N/A	N/A	N/A	**0.94**	0.94	0.94	0.65	0.65	0.65
RF00489	**0.96**	0.92	1.00	0.92	0.85	1.00	**0.96**	0.92	1.00	**0.96**	0.92	1.00
RF00113	0.85	0.83	0.87	0.79	0.79	0.79	**0.98**	0.96	1.00	**0.98**	0.96	1.00

Mean	0.86	0.83	0.89	0.88	0.84	0.94	0.89	0.85	0.93	**0.94**	0.92	0.96
Std. Dev.	0.24	0.25	0.24	0.15	0.18	0.12	0.23	0.24	0.23	**0.08**	0.09	0.08

The best MCC value for each family, and the best mean and standard deviation are highlighted in bold. Note that RSpredict has the best standard deviation in MCC, SN and SL respectively compared with the other three tools. This shows that RSpredict has stable performance across all the families tested in the experiment.
